# The Yeast Fermentation Effect on Content of Bioactive, Nutritional and Anti-Nutritional Factors in Rapeseed Meal

**DOI:** 10.3390/foods11192972

**Published:** 2022-09-23

**Authors:** Mihaela Vlassa, Miuța Filip, Ionelia Țăranu, Daniela Marin, Arabela Elena Untea, Mariana Ropotă, Cătălin Dragomir, Mihaela Sărăcilă

**Affiliations:** 1 Raluca Ripan Institute for Research in Chemistry, Babeș-Bolyai University, 400294 Cluj-Napoca, Romania; 2 National Institute for Research and Development for Biology and Animal Nutrition, 077015 Ilfov, Romania

**Keywords:** rapeseed meal, fermentation, yeasts, anti-nutritive factors, bioactive compounds

## Abstract

The aim of this study was to evaluate the changes in the content of bioactive, nutritional and anti-nutritional factors in rapeseed meal that was fermented with *Saccharomyces cerevisiae* or *Saccharomyces boulardii* yeasts at two different periods of time, for improvement of nutritional characteristics in piglets’ feeding. The fermentation has reduced the content of two anti-nutritional factors, intact glucosinolates and 3-butyl isothiocyanate, by 51.60–66.04% and 55.21–63.39%, respectively, by fermentation with either *Saccharomyces cerevisiae* or *Saccharomyces boulardii* for 24 h. The fermentation by these yeasts also lowered the content of total polyphenolic compounds by 21.58–23.55% and antioxidant activity (DPPH) by 17.03–21.07%. Furthermore, the content of carbohydrates and organic acids has dramatically decreased between 89.20 and 98.35% and between 31.48 and 77.18%, respectively. However, the content of some individual phenolic acids (gallic, *p*-coumaric, sinapic) and crude protein content (10–13%) has been increased. Thus, the results showed that fermentation with *Saccharomyces cerevisiae* or *Saccharomyces boulardii* has reduced the content of antinutritive factors and increased the protein content of the rapeseed meal, without major adverse effects on its overall nutritive value.

## 1. Introduction

The formulation of farm animals’ diets focuses on the supply of the main nutrients, but also the bioactive compounds of the dietary ingredients can have an impact on an animal’s health and performance [1]. Extensive studies of rapeseed varieties (whole seeds, oils or the meals obtained from the oil extraction) revealed that they are also rich sources of natural components, having various properties such as antioxidant [2] or anti-nutritional [3]. 

Rapeseed meal is a commonly used protein ingredient for animal feeding and its content of fibres and carbohydrates, phenolic compounds, flavonoids and minerals complete the overall nutritional value of the diets [4,5]. Analytical studies showed a high level of polyphenolic compounds in rapeseed meal with high antioxidant/radical scavenging properties estimated to be about five times higher than its major competitor, soybean meal [6,7]. Among the rapeseed polyphenols, recognized as powerful antioxidants are sinapic acid and sinapine, intensively investigated for the development of nutraceutical, food or pharmaceutical products, especially useful in the treatment of neurodegenerative diseases [7,8,9]. In rapeseed, the phenolic compounds fractionated into different groups, including free phenolic acids (gallic, chlorogenic, cinnamic, sinapic, ferulic, syringic, chlorogenic, ellagic, *p*-coumaric, vanillic, caffeic, gallic, *p*-hydroxybenzoic acids) are natural antioxidants, with important functions in the prevention and treatment of some chronic diseases, such as heart, neurodegenerative, aging, cancer or rheumatoid arthritis [10,11,12,13]. The antioxidant activities of the polyphenolic compounds of rapeseed meal and crude oil are correlated with their concentrations [10].

Generally, rapeseed meal has a positive influence on growth performance, blood profiles, nutrient digestibility and economic benefit of growing-finishing pigs [14]. However, the nutrient digestibility and valorisation of rapeseed meal is lowered by the presence of anti-nutritional factors, such as tannins, phytic acid and glucosinolates, whose content should be reduced [15] through plant breeding programs or through other means. However, for tannins, the reduction is less important due to the fact that tannins that are found in rapeseed meal/canola are water-insoluble compounds and are located in the hulls and by consequence, have a minimal effect on the nutritive value of the rapeseed. Importantly, nutritional studies demonstrated some beneficial effects of tannins on performance (feed efficiency) and the intestinal ecosystem in weaning piglets [16]. The problem remains for glucosinolates. Fermentation of the rapeseed meal can solve this problem; according to [17], it may lead to the decrease in glucosinolates, isothiocyanate, tannin and phytic acid levels. The use of fermented rapeseed meal in compound feeds for sows is an effective way to reduce the anti-nutrient (phytate phosphate and glucosinolates) levels and to increase the level of lactic acid, as well as to stimulate the immune system, which improves the health of piglets [18]. Organic acids, which can be increased through fermentation, are known to be effective food and feed preservatives and also, to have beneficial effects on animals’ performances. The inclusion of organic acids (formic, lactic, sorbic, fumaric, citric and malic acid) in the diet of weanling pigs led to significant improvements in growth rate, associated with positive effects on reducing gastric pH, preventing the growth of pathogens, acting as an energy source during gastrointestinal tract intermediary metabolism, increasing apparent total tract digestibility and improving growth performance [19]. 

Most of the previous studies focusing on the valorisation of fermented rapeseed in pigs’ diets refer to fermentation with lactic acid bacteria [20] or with *Bacillus clausii* and *Saccharomyces cariocanus* strains, with the main goal being to reduce the content of anti-nutritional factors [21] or using *Aspergillus niger* fermentation to improve the nutritional quality of rapeseed meal, but altering chemical composition and physicochemical properties [22]. Lactic acid bacteria (LAB; *Lactobacillus plantarum* and *Bacillus clausii*) and yeasts (*Saccharomyces cariocanus* and *Wickerhamomyces anomalus*) were used for degrading free gossypol and glucosinolate in the fermented rapeseed meal and to improve the utilization efficiency of these protein sources [23]. To our knowledge, there are very few studies which have investigated the fermentation of other microorganisms, such as yeasts. For example, *Saccharomyces cerevisiae* was used for fermenting the rapeseed meal to be used in fish diets, but the references to fermented rapeseed meal as an ingredient for pig diets are scarce [24]. Recent studies show that *Saccharomyces boulardii* probiotic yeast with beneficial health effects that have been studied regarding its potential for use in the development of innovative functional foods [25]. Literature data show that *Saccharomyces boulardii* yeast has not been used before in the fermentation of rapeseed meal. Based on these findings, our study aims to use these types of yeasts for fermenting rapeseed meal, in order to improve it nutritional properties of meal.

Therefore, the aim of this study was to assess the effects of the rapeseed meal’s fermentation with *Saccharomyces cerevisiae* or *Saccharomyces boulardii* yeasts on the content of anti-nutritional factors such as intact glusosinolates and 3-butyl isothiocyanate, as well as on the content of bioactive compounds, antioxidant activity (DPPH, ABTS assays, Fe^2+^ chelating activity) and total phenolic content (TPC). The study also focused on the effects of fermentation on individual polyphenolic compounds, carbohydrates, organic acids, fatty acids and minerals of the fermented rapeseed meal.

## 2. Materials and Methods

### 2.1. Rapeseed Meal Samples

The rapeseed meal used in this study was provided by EXPUR S.A., Slobozia, Romania and the samples were used as unfermented rapeseed meal (RSM), and either rapeseed meal fermented with *Saccharomyces cerevisiae* (EC 1118–commercial yeast for vinification) for 24 h or 72h (FRSM-*SC,* 24 h, 72 h) or rapeseed meal fermented with *Saccharomyces boulardii* (CNCM I-745) for 24 h and 72 h (FRSM-*SB*, 24 h, 72 h). The fermented samples were prepared in duplicates. 

The fermentation process was carried out by using the methods described by [26] in the following steps: (1) preparation of inoculum medium and fermentation medium; (2) inoculation of medium with yeasts; (3) inoculation of fermentation medium with the inoculum obtained in step 2, followed by incubation at 24 h, 130 rpm and 27 °C; (4) inoculation of RSM with the inoculum obtained in step 3, followed by solid-state fermentation in plastic bags. Two periods of fermentation (24 h, 72 h) were tested, according to the increase in active compounds concentration; and (5) processing of FRSM by grinding (40-mesh sieve) for related index determination. After fermentation, the samples were dried in the oven at 60 °C.

### 2.2. Chemicals and Materials

The acetonitrile, methanol and hexane with HPLC grade, sulfuric acid, formic acid and organic acids standards (tartaric, citric, malic, succinic and oxalic) and fatty acid methyl esters standard mixture (FAME) were purchased from Merck (Darmstadt, Germany), The standards of flavonoids (catechin, epicatechin, rutin, quercetin and luteolin), phenolic acids (gallic, vanillic, caffeic, *p*-coumaric, ferulic and sinapic), carbohydrates (glucose, fructose, sucrose, maltose), citrate-phosphate and sinigrin were purchased from Sigma-Aldrich Co. (St. Louis, MO, USA). The 2,2′-Diphenyl-1-picrylhydrazyl radical (DPPH, 95%) and 2,2’-Azino-bis-(3-ethylbenzothiazoline-6-sulfonic acid) diammonium salt, (ABTS, 98%) were purchased from Alpha Aesar, Thermo Fisher (Kandel, Germany). 6-hydroxy-2,5,7,8-tetramethylchroman-2-carboxylic acid (Trolox, 98%) was purchased from TCI (Portland, OR, USA). The 3-butenyl isothiocyanate standard was purchased from Chem Cruz Biotechnology, Inc. The myrosinase was extracted from rapeseed [27].

### 2.3. Determination of Proximate Chemical Composition of the Rapeseed Meal Samples

The proximate chemical composition was performed by the WEENDE method (Regulation (EC) No 152/2009) which included the determination of the dry matter, crude protein, crude fat, crude cellulose and ash. The crude protein was determined according to the standard SR EN ISO 5983-2:2009 AOAC2001.11 based on the determination of the nitrogen content (Kjeldahl principle). The crude fat was determined according to the standard SR ISO 6492:2001 based on the extraction of fat in organic solvents (ethyl ether, petroleum ether, etc.), drying and weighing of the residue. The ash was determined by the calcination temperature of 600 °C according to standard SR EN ISO2171: 2010. The gravimetric method was used for ash according to the recommendations of Regulation (EC) no. 152/2009 SR EN ISO 2171:2010 using a Nabertherm calcination furnace (Nabertherm GmbH, Lilienthal, Germany).

### 2.4. Samples Preparation

*The extraction of intact glucosinolates*. An aliquot of the ground sample (100 mg) mixed with water (1 mL) was well stirred and heated at 90–92 °C for 15 min. The sample was then sonicated (Bandelin Sonorex, Berlin, Germany) at ultrasonic power 100% and 80 KHz ultrasonic frequency, for 15 minutes, centrifuged at 4500 rpm for 10 min and the clear supernatant was collected after repeating the extraction procedure 2 more times.

*Sample preparation for 3-butenyl isothiocyanate determination.* To an aliquot of the sample (200 mg), 2 mL of 80% methanol solution was added, well stirred and heated at 90–92 °C for 5 min, then sonicated at ultrasonic power 100% and 80 KHz ultrasonic frequency for 15 min and centrifuged at 4500 rpm (Eppendorf Centrifuge 5804 R, Germany) for 10 min and collected in the supernatant (repeated 2 more times). The collected solutions were evaporated to dryness and the residue was treated with a citrate-phosphate buffer pH 6, and myrosinase and the hexane are added and left to react for 12 h at a 37 °C temperature.

*Extraction of phenolic compounds for determination of total phenolic content (TPC) and antioxidant activity.* About 0.5 g of the ground sample and 10 mL of methanol-water (1:1 *v/v*) were sonicated at ultrasonic power 100% and 80 KHz ultrasonic frequency, at room temperature for 60 min, then centrifuged at 4500 rpm for 20 min. All samples were analysed in triplicate.

*Preparation of the extracts of individual phenolic compounds, carbohydrates and organic acids.* Extraction of carbohydrates and organic acids from the studied samples was carried out in Millipore water, while the individual phenolic compounds were extracted in 80% methanol solution. Then, 1 g of the sample with 10 mL of extraction solvent was well stirred, sonicated for 60 minutes at ultrasonic power 100% and 80 KHz ultrasonic frequency, centrifuged at 4500 rpm for 20 min, and then the supernatant was filtered through a 0.45 μm syringe filter and injected into HPLC.

*Sample preparation for fatty acids determination.* Fatty acids were prepared from the total lipid extracts and were converted to their methyl esters by transesterification in methanol containing 3% concentrated sulfuric acid at 80 °C for 4 h. Methyl esters of fatty acids were analysed by Gas Chromatography. 

### 2.5. HPLC Determination of Intact Glucosinolates and 3-Butenyl Isothiocyanate

The analyses of anti-nutritional compounds, the intact glucosinolates and 3-butenyl isothiocyanate, were performed on HPLC Agilent Technologies 1200 Series, (Agilent Technologies, Morge, Switzerland), equipped with a DAD detector. The chromatographic data were collected and processed using Chem Station software (version B.04.01, Waldbronn, Germany). 

Separation and identification of anti-nutritional compounds were performed on a Phenomenex Luna Omega chromatographic column (C18 100 Å, 5 µm, 250 × 4.6 mm) at 25 °C. The HPLC profiles of intact glucosinolates (expressed as sinigrin) from rapeseed meal samples were obtained by the method adapted from [28]. The elution of glucosinolates was performed with 0.2 M ammonium sulphate solution at 1 mL/min flow rate, 20 µL volume injection and detection at 227 nm. The results are expressed as mg sinigrin/g dry sample. The HPLC profiles of 3-butyl isothiocyanate were analysed by the modified method of [27]. The elution of 3-butenyl isothiocyanate was performed with acetonitrile-water (1:1, *v*/*v*) at a flow rate of 1 mL/min and detection at 247 nm. 

### 2.6. Analysis of Total Phenolic Compounds (TPC)

TPC was determined spectrophotometrically using UV-Vis Spectrophotometer Specord 205 (Analytik Jena, GMbH, Germany) and Folin-Ciocalteu (FC) reagent, according to the procedure described previously [29] that was slightly modified. 

Briefly, 0.4 mL of methanolic RSM extract and 2 mL of FC reagent (diluted 1:1) were shaken for 3 min and 1.6 mL of sodium carbonate solution (7.5%) was added and brought to 10 mL, using water. After 10 minutes at 50 °C, solutions were cooled, and the absorbance was measured at 760 nm against a reagent blank (0.4 mL water + 2 mL of FC reagent + 1.6 mL sodium carbonate solution). The absorbance of gallic acid (GAE) standards of the samples were recorded. The TPC of each rapeseed extract was quantified as mg gallic acid equivalent per 100 g dry weight (mg GAE/100 g). All determinations were performed in triplicates. 

### 2.7. Determination of Antioxidant Activity

Three different chemical methods namely DPPH (2,2′-diphenyl-1-picrylhydrazyl radical) and ABTS (2,2′-azinobis-(3-ethylbenzothiazoline-6-sulfonate) assays and iron (Fe^2+^ ) chelating activity were used for evaluating the antioxidant activity of the studied samples. All determinations were performed in triplicate.

*DPPH• radical scavenging assay.* The adapted DPPH method [10] was used for the spectrophotometrically determination (517 nm) against methanol as the blank of the antioxidant capacity of the studied samples. The free radical scavenging activity of the extracts was measured by absorbance (Abs) with respect to the effect of standard solutions of methanolic Trolox (6-hydroxy-2,5,7,8-tetramethylchroman-2-carboxylic acid) (0.02–0.1 μmol/mL) or rapeseed meal extracts on the DPPH• radical, according to the following procedure: An aliquot of 0.5 mL of each Trolox solution (or extract) was added to 2 mL methanol and 0.5 mL DPPH solution. The control sample was prepared by mixing 2.5 mL methanol with 0.5 mL of DPPH solution. 

The scavenging activity of DPPH was calculated as follows (Equation (1)):

(1) $$\mathrm{DPPH}\;\mathrm{scavenging}\;\mathrm{activity}\;\left(\%\right)=\frac{\left(\mathrm{Abs}\;\mathrm{control}\;-\;\mathrm{Abs}\;\mathrm{sample}\right)}{\mathrm{Abs}\;\mathrm{control}}\times 100$$

The effective concentrations (DPPH) were expressed in µmol Trolox/100g dry weight.

*ABTS•+ radical scavenging assay.* The spectrophotometric determination of the antioxidant activity of samples by the ABTS method is based on the percentage inhibition of peroxidation of this radical. The reaction was carried out according to a previously described method [30], with modifications. The radical cation ABTS•+ was generated by persulfate oxidation of ABTS [11]. 

The working solution (7 mM ABTS and 2.45 mM potassium persulfate) in equal quantities allowed them to react for 17 h, at room temperature, in the dark. This solution was then diluted with methanol to obtain an absorbance between 0.700 to 0.800 units at 734 nm. Rapeseed meal extracts (0.5 mL) are allowed to react with 2.5 mL of the fresh ABTS solution time of 6 min. 

The ABTS scavenging activity of the extracts was measured taking into account the effect of the Trolox standard solutions (2.5–12.5 µg/mL), regarding the discolouring capacity of the blue-green colour of the ABTS solution. The percentage inhibition was calculated using Equation (2):

(2) $$\mathrm{ABTS}•+\;\mathrm{radical}\;\mathrm{scavenging}\;\mathrm{activity}\;\left(\%\right)=\frac{\mathrm{Abs}\;\mathrm{control}-\mathrm{Abs}\;\mathrm{sample}}{\mathrm{Abs}\;\mathrm{control}}\times 100$$

where: Abs control is the absorbance of ABTS•+ radical in methanol; Abs sample is the absorbance of ABTS•+ radical solution mixed with sample extract/standard. 

The effective concentrations (ABTS) were expressed in µmol Trolox/100 g dry weight.

*Iron (Fe^2+^) chelating activity.* The chelating effect on ferrous ions was determined according to the literature methods [31,32] with slight modifications. The method was based upon the ability of the extract to compete with ferrozine for ferrous ions. Briefly, an exact volume of 1 mL of methanolic rapeseed extract (1:10, *w*/*v*) and 1.6 mL deionised water was placed in a 10 mL volumetric flask and mixed well. Then, 0.06 mL of 2 mM FeCl_2_ solution was added in each tube and left to react for 3 min, when 0.12 mL of 5 mM ferrozine solution was added. The mixture was shaken vigorously and left at room temperature for 10 min. The absorbance of the purple colour of the formed complex was measured against blank at 562 nm using a UV-VIS spectrophotometer (JASCO V 560). A decrease in absorbance corresponds to an increase in iron chelating ability. The Iron (Fe^2+^) chelating activity can be expressed either as a percentage of inhibition (Fe^2+^ chelating activity%) or as mg disodium ethylenediamine tetra-acetic acid (EDTA-Na_2_) equivalents per g sample (equiv. mg EDTA/g). 

The percentage of Fe^2+^-ferrozine complex formation was calculated according to Equation (3) given below:

(3) $${\mathrm{Fe}}^{2+}\;\mathrm{chelating}\;\mathrm{activity}\;(\%)=\left[1-\frac{A_{s}}{A_{b}}\right]\times 100$$

where A_s_ = absorbance of sample and A_b_ = absorbance of blank solution.

Six concentrations of disodium ethylenediamine tetra-acetic acid (EDTA-Na_2_) were used to plot an analytical curve (R^2^ = 0.9972). 

### 2.8. HPLC Determination of the Individual Phenolic Compounds, Carbohydrates and Organic Acids

The analyses of individual phenolic compounds (flavonoids and phenolic acids), carbohydrates and organic acids were carried out by high-performance liquid chromatography (HPLC) on a Jasco Chromatograph (Jasco Corporation, Tokyo, Japan) equipped with UV/Vis detector, Refractive Index detector and an injection valve equipped with a 20 μL sample loop (Rheodyne). The ChromPass software was used to control the HPLC system and to collect and process the chromatographic data. 

Determination of *individual phenolic compounds (flavonoids and phenolic acids)* was carried out using the HPLC gradient analysis method described by [33]. Separation of flavonoids and phenolic acids were carried out on the Lichrosorb RP-C18 column (25 × 0.46 cm) at 22 °C column temperature and UV detection at 270 nm. The mobile phase was a mixture of methanol (A, HPLC grade) and 0.1% formic acid solution (Millipore ultrapure water) and a gradient was applied according to the following method: 0–10 min, linear gradient 10–25% A; 10–25 min, linear gradient 25–30% A; 25–50 min, linear gradient 35–50% A; 50–70 min, isocratic 50% A. The flow rate was 1 mL/min. 

The *carbohydrate* content was determined by the HPLC-RI method, adapted from [34]. The chromatographic separation was carried out on the Kromasil-NH_2_ (250 × 4.6 mm) column and elution with acetonitrile–water, 70:30, *v*/*v* as the mobile phase, flow rate 1 mL/min and column temperature 25 °C and RI detection.

The quantification of *organic acids* was performed by a method presented by [35]. Separation was carried out on a CarboSep Coregel 87H3 column (300 × 7.8 mm), at 35 °C column temperature. The mobile phase was the 0.005M sulfuric acid solution. The flow rate was 1 mL/min and UV detection was 214 nm.

### 2.9. Determination of Fatty Acids Profile

Fatty acid content was assessed by gas chromatography according to ISO/TS 17764–2 (2008), as described by [36]. Briefly, fatty acids from the total lipid extracts were converted to their methyl esters by transesterification in methanol containing 3% concentrated sulfuric acid at 80 °C for 4 h. Methyl esters of fatty acids were analysed in a Perkin Elmer-Clarus 500 chromatograph (PerkinElmer Inc., Shelton, DC, USA) equipped with flame ionization detector (FID) and fitted with a BPX70 capillary column (60 m × 0.25 mm i.d., 0.25 μm film thickness). The column temperature was programmed at 5 °C/min from 180 °C to 220 °C and the temperatures for the injector and detector were 250 °C and 260 °C, respectively. The carrier gas was hydrogen (35 cm/s linear velocity at 180 °C) and the splitting ratio was 1:100. Fatty acid identification was performed by comparison with retention times of the known standards. The results were expressed as g fatty acid/100 g total fatty acid methyl esters (FAME). The studied fatty acids were saturated fatty acids (SFA), monounsaturated fatty acids (MUFA), polyunsaturated fatty acids (PUFA) and unsaturated fatty acids (UFA).

### 2.10. Determination of Minerals

The concentration of mineral macro-elements Calcium (Ca), Natrium (Na), Potassium (K) and Magnesium (Mg) was determined by atomic absorption spectrometry (AAS) according to the standard SR ISO 6490-2:1983 for Ca and SR ISO 7485:2000 for Na, K and Mg using an atomic absorption spectrophotometer Thermo Electron (Thermo Fisher Scientific Inc., Göteborg, Sweden). The concentration of micro-minerals Copper (Cu), Iron (Fe), Zinc (Zn) and Manganese (Mn) was determined by AAS after microwave digestion and mineralization with nitric acid (Regulation (EC) no. 152/2009 STAS 9597/16-86STAS 9597/17-86). Working parameters: wavelength (nm): 324.8 (Cu), 279.5 (Mn), 213.9 (Zn); bandpass (nm): 0.5 (Cu), 0.2 (Mn), and 0.5 (Zn); lamp current (mA): 5 (Cu), 12 (Mn), 10 (Zn).

### 2.11. Statistical Analysis

The effects of the experimental factors, time (24, 72 h) and type of yeast (no yeast, *Saccharomyces cerevisiae* and *Saccharomyces boulardii*) were assessed using the GLM procedure (Mintab 16.0 Statistical Software, State College, PA, USA), followed by the Tuckey test in order to differentiate among experimental treatments. The statistical difference was declared at *p* < 0.05. The Tuckey test was used to assess data variability by calculating the standard error of the means corresponding to the levels of each of the two experimental factors, “time” (0, 24, 72 h) and yeast (no yeast, Sc, Sb).

## 3. Results

### 3.1. Effect of the Fermentation on the Anti-Nutritional Compounds of the Rapeseed Meal

The content of these anti-nutritional factors, before and after the fermentations, are presented in Table 1.

Fermentation for 24 h, either with *Saccharomyces cerevisiae* or *Saccharomyces boulardii,* led to significant decreases (*p* < 0.001) in intact glucosinolates (by 51.60% and 66.04%, respectively), while further fermentation for another 48 h brought no further reductions. The same magnitude of the decrease (*p* < 0.001) was observed in the case of 3-butenyl isothiocyanate, by 55.21 or 63.40%, following the 24 h fermentation with *Saccharomyces cerevisiae* or *Saccharomyces boulardii*, respectively. Prolongation of the fermentation also brought no significant supplementary reduction. 

### 3.2. Effects of the Fermentation on the Proximate Chemical Composition of the Rapeseed Meal

The proximate chemical composition of the rapeseed meal, before and after the fermentations, is presented in Table 2.

Our results showed a significantly higher (*p* < 0.001) crude protein content of the FRSM, either with *Saccharomyces cerevisiae* or *Saccharomyces boulardii*, for both fermentation periods (24 or 72 h) +13.12% and +11.17% for *Saccharomyces cerevisiae* fermentation and +11.67% and +10.06% for *Saccharomyces boulardii,* respectively; compared to RSM, it is important to underline that the fermentation for more than 24 h brings no further advantages from the point of view of protein content. The increase in the crude protein content was also observed when fermenting canola meal (a variety of rapeseed meal) with *Saccharomyces cerevisiae* for 24 h [37]. An increase in the protein content was reported following the fermentation of rapeseed meal with *Rhizopus oligosporus*. A 10% increase in protein content is important from the point of view of diet optimisation, as it brings the nutritive value of rapeseed meal closer to the value of soybean meal [38].

The overall decrease in the crude fat content, although statistically significant, is less relevant as the overall content is very low and therefore without significant influence on the dietary energy supply. The content of crude cellulose significantly increased in all the fermented samples, disregarding the utilised yeast or the duration of the fermentation. In literature, the effects of the fermentation on the crude fibre content of rapeseed meal are contradictory: from a significant decrease [17,22] to a noticeable increase [37,39]. According to these authors, this depends on the fermentation conditions and on the ability or inability of the microorganisms utilised for fermentations to degrade various constituents of the rapeseed meal (fibers, non-structural carbohydrates) but also on the processing conditions. The content of the yeast biomass in soluble fibre is also to be taken into account [40].

Although some differences among treatments were observed for DM and for the ash, no clear conclusion on the effects of either yeasts or length of fermentation can be drawn.

### 3.3. Fermentation Effect on the Content of the Bioactive and Other Nutritional Compounds in the Rapeseed Meal Samples

#### 3.3.1. Effects of Fermentation on Phenolic Compounds and Antioxidant Activity

The results regarding total phenolic content (TPC), antioxidant activity (DPPH, ABTS) and Fe^2+^ chelating activity in studied samples were shown in Table 3. 

It can be noted that the fermentation effect on the total phenolic compounds and the antioxidant activity expressed by DPPH, followed the same pattern as in the case of glucosinolates, a strong and statistically significant decrease (*p* < 0.001) of 21.58–23.55% and 17.03–21.07%, respectively, disregarding the utilised yeast and the period of fermentation. This consolidates the idea that a 24 h fermentation is enough to obtain the desired effects. 

However, in the case of the antioxidant activity expressed by ABTS, the effect of fermentation at 24 h led to a significant decrease (*p* < 0.001) of 15.71% and 15.56% at FRSM with *Saccharomyces cerevisiae* and *Saccharomyces boulardii*, respectively. At 72 h, fermentation led to a significant diminution (*p* < 0.001) of 20.65% for FRSM with *Saccharomyces cerevisiae* and 28.16% for FRSM with *Saccharomyces boulardii*.

The Fe^2+^ chelating activity also showed a statistically significant increase (*p* < 0.001) of 34.45% for FRSM with *Saccharomyces cerevisiae* and 40.93% FRSM with *Saccharomyces boulardii* after 24 h fermentation time. After 72 h fermentation time, the results were 24.11% FRSM with *Saccharomyces cerevisiae* and 23.06% FRSM with *Saccharomyces boulardii*. These results indicated that FRSM samples displayed higher ferrous-ion chelating activity compared to RSM samples.

#### 3.3.2. Effects of Fermentation on Individual Phenolic Compounds

The individual phenolic compounds (phenolic acids: gallic, vanillic, caffeic, *p*-coumaric, ferulic, sinapic and flavonoids: catechin, epicatechin, rutin, quercetin and luteolin) of the RSM and FRSM is presented in Table 4.

The effect of the fermentation on the sum of the individual phenolic compounds (*p* < 0.001) determines a decrease between 34 and 52%, depending on the fermentation time and utilised yeast. This decrease is a positive result because it was stipulated that a high concentration of polyphenols in rapeseed meal limits the digestibility and bioavailability of protein and has an impact on the flavor and, by consequence, on the taste of feed [6]. 

A positive result is also the fact that the fermentation determined a very high and significant increase in sinapic acid (*p* < 0.001) considered a specific polyphenol for rapeseed in all fermentation conditions (5.3–7.6 times the control level). The results showed a significant increase in gallic acid (*p* < 0.001) (both yeasts and times) except in fermentation with *Saccharomyces boulardii* (72 h) and in *p*-coumaric acid (both yeasts and times), which is a precursor of resveratrol synthesis.

### 3.4. Fermentation Effects on Carbohydrates and Organic Acids Content

It is commonly acknowledged that the fermentation of rapeseed meal is a process that leads to the consumption of non-structural, particularly soluble carbohydrates. The influence of the fermentation process and conditions (yeast type and fermentation time) on the content of the individual carbohydrates in rapeseed meal is presented in Table 5.

Predictably, fermentation in the presence of yeasts led to a dramatic decrease in the soluble carbohydrates. An overall image is given by the total soluble carbohydrates, which significantly decreased (*p* < 0.001) with values between 89.20 and 98.35%. Expectedly, a longer fermentation time led to a further decrease in the soluble carbohydrate content. On the other hand, the decrease was more pronounced when fermentation was done with *Saccharomyces boulardii*, compared to *Saccharomyces cerevisiae*, especially in the case of sucrose and maltose.

The influence of the fermentation effect on the content of the organic acids in rapeseed meal is presented in Table 6. 

The overall contents of the organic acids were significantly reduced (*p* < 0.001) by the fermentation, although to a lower extent than in the case of soluble carbohydrates. The magnitude of these reductions was dependent on the fermentation time (72 h versus 24 h), as the fermentation for 48 more hours brought the overall decrease from 31.48% to 77.18%. The reduction was also more pronounced when the fermentation process was done with *Saccharomyces boulardii*, compared to *Saccharomyces cerevisiae*, particularly in the case of tartaric acid that almost disappeared in the samples fermented with *Saccharomyces boulardii*. An exception was recorded in the case of succinic acid, which has increased (*p* < 0.001) several times (4–7 times), for both yeasts. 

### 3.5. Fermentation Effects on Fatty Acids Concentration

The fermentation effects on fatty acid concentration in rapeseed meal are presented in Table 7. 

Chromatographic analysis revealed that fermentation of rapeseed led to a tendency of increasing the relative proportion of UFA (g/100 g FAME), from 85.83 in the unfermented samples to 86.52–87.43 in the fermented samples. This was mainly determined by the significant increase (*p* < 0.001) in the MUFA content (g/100 g FAME) from 42.15 to 46.3–47.81, as the effects on PUFA content (g/100 g FAME) were negative (decreasing from 43.68 to 38.71–40.2). The 10–11% increase in MUFA balanced the 7–12% decrease in PUFA; thus, leading to a slight increase in UFA in fermented versus unfermented rapeseed meal. 

The most important concentration was recorded for *cis*-oleic acid (omega-9) (g/100 g FAME) of 45.88 at 24 h and 45.10 at 72 h for rapeseed meal fermented with *Saccharomyces cerevisiae* and of 44.49 at 24 h and 44.51 at 72 h for FRSM with *Saccharomyces boulardi* compared to unfermented RSM of 41.20 g/100 g FAME.

### 3.6. Fermentation Effects on Minerals Concentration

The result of the mineral analyses showed that the fermentation, regardless of the *Saccharomyces* strain and the fermentation time, did not improve the concentration of macro-elements in the rapeseed meal (Table 8). 

There were no significant differences, except K mineral, in which case the effects were inconsistent; no significant effect in the case of fermentation with *Saccharomyces cerevisiae* and numerical or even significant decrease in the case of fermentation with *Saccharomyces boulardii* (at 24- and 72-h fermentation, respectively).

Concerning the micro-elements, the fermentation doubled the Cu concentration, from 10.31 ppm in unfermented RSM to 23.97 ppm in the samples of FRSM (overall average) and also significantly increased (*p* < 0.001) the Zn concentration from 117.70 ppm to 138.90 ppm (overall average). 

## 4. Discussion

### 4.1. Effect of the Fermentation on the Anti-Nutritional Compounds of the Rapeseed Meal

In general, solid-state fermentation has been reported to be an effective way to reduce undesired substances of rapeseed meal including phytic acid, glucosinolates, tannins, etc., [41]. The magnitude of the decrease obtained in our study is in line with those obtained by [38], in rapeseed meal fermented with *Rhizopus oligosporus* (−43.1%). They also obtained decreases in thiooxazolidones and phytic acid, by 34% and 42.4%, respectively. 

In our case, a strong decrease in intact glucosinolates (*p* < 0.001) by 51.60% (fermentation for 24 h with *Saccharomyces cerevisiae*) and 66.04% (fermentation for 24 h with *Saccharomyces boulardii*), respectively, were obtained. 

Such a major decrease in glucosinolates is important from a nutritional point of view, especially for monogastric animals, even in the case of the rapeseed cultivars that are bred for low anti-nutritive factors. Glucosinolates are secondary metabolites derived from amino acids produced by plants belonging to the genus *Brassica*. Chemically, they are sulfate anions that appear as glycosides. To date, over 90 different glucosinolates have been reported for this genus [42] and structurally, they can be classified into three major classes, namely: aliphatic, idolyl and arylalkyl glucosinolates. Some of the aliphatic glucosinolates are the most abundant (allyl glucosinolate, 3-butenyl glucosinolate, 2-hidroxy-3-butenyl glucosinolate) and are responsible for the presence of spicy flavours in crucifers due to thio- and isothiocyanates released after their hydrolysis. As the goitrogenic properties of glucosinolates are attributed to high levels of thio- and isothiocyanates [43], the elimination of nutritionally toxic glucosinolates continues to be a challenge.

Depending on the reaction conditions, the breakdown of glucosinolates may be enzymatic and/or non-enzymatic; as myrosinase is present alongside glucosinolates in plant tissue, the first process predominates, with chemical hydrolysis only functioning if myrosinase is inactivated, for example, after cooking or under extremely acidic/basic conditions. Iso enzymes myrosinase and glucosinolates are localized in all cells, but are compartmentalized; glucosinolates are stored in vacuol and myrosinases are located in the cytoplasm. After tissue damage, the enzyme and substrate come into contact, causing glucose hydrolysis and thus producing glucose and an unstable aglycone that spontaneously undergoes rearrangement to an isothiocyanate or nitrile and elemental sulphur. The goitrin and isothiocyanates synthesized via myrosinase mediated hydrolysis of its constituent glucosinolates limits the utilisation of rapeseed meal [27]. 

In our case, we observed an important decrease in 3-butenyl isothiocyanate (*p* < 0.001) by 55.21% (24 h fermentation with *Saccharomyces cerevisiae*) and 63.39% (24 h fermentation with *Saccharomyces boulardii*), respectively.

The analysis of these compounds allows for an exhaustive nutritional evaluation of studied rapeseed meals in swine feed. Prolongation of the fermentation also brought no significant supplementary reduction. 

### 4.2. Effects of the Fermentation on the Proximate Chemical Composition of the Rapeseed Meal

The increase in the crude protein content was also observed by [37] when fermenting canola meal (a variety of rapeseed meal) with *Saccharomyces cerevisiae* for 24 h. Study [38] also reported an increase in the protein content following the fermentation of rapeseed meal with *Rhizopus oligosporus.* A 10% increase in protein content is important from the point of view of diet optimisation, as it brings the nutritional value of rapeseed meal closer to the value of soybean meal. 

The overall decrease in the crude fat content, although statistically significant, is less relevant as the overall content is very low and therefore, without significant influence on the dietary energy supply. The content of crude cellulose significantly increased in all the fermented samples, disregarding the utilised yeast or the duration of the fermentation. In literature, the effects of the fermentation on the crude fibre content of the rapeseed meal are contradictory: from a significant decrease of 24.68% [17] and 28.21% [22], to a noticeable increase of 9.2% [37] and 72.86% [39] in fermented rapeseed meal. According to these authors, this depends on the fermentation conditions and on the ability or inability of the microorganisms utilised for fermentations to degrade various constituents of the rapeseed meal (fibers, non-structural carbohydrates) but also on the processing conditions. The content of the yeast biomass in soluble fibre is also to be taken into account [40].

### 4.3. Effects of Fermentation on Phenolic Compounds and Antioxidant Activity

Plant bioactive phenolics include flavonoids, phenolic acids, coumarins, stilbenes, hydrolysable and condensed tannins, lignans and lignins. They are antioxidants acting in different mechanisms, such as: scavenging of free radicals, inhibition of oxidative enzymes, chelation of transition metals or due to interaction with bio membranes [44].

In our case, the fermentation effect on the total phenolic content and the antioxidant activity expressed by DPPH were statistically (*p* < 0.001) significantly decreased.

Similar results were reported by [45], that studied the fermentation of rapeseed pressed cake with tempeh mould Rhizopus and its effect on various components, including the phenolics. Similar values for DPPH and ABTS were also found in various rapeseed cultivars by [46]. The phenolic compounds are not the only compounds with antioxidant potential in the studied rapeseed extracts. However, the phenolic content is an important factor in determining the antioxidant capacity of rapeseeds [10]. The Fe^2+^ ion-chelating activity of FRSM displayed a higher value compared to the RSM. The comparative results of ferrous-ion-chelating activity were reported in literature for fermented rapeseed meal with protease [47].

However, the effects on individual phenolic compounds are divergent. On one hand, the results showed a significant increase in gallic acid (both yeasts and times excepting fermentation with *Saccharomyces boulardii*, 72 h), *p*-coumaric acid (both yeasts and times) and phenolic compounds with the capacity in modulating the pro- and anti-inflammatory, as well as the anti-oxidative stress effect in blood and the nervous system included [48,49,50]. Interestingly, the fermentation determined a very high and significant increase in sinapic acid, which is considered a specific polyphenol for rapeseed in all fermentation conditions (5.3–7.6 times the control level). In rapeseed meal, the sinapic acid is found in esterified forms [51], mainly sinapine (sinapoyl choline) and glucopyranosyl sinapate. and the amount of sinapic acid in the free form does not exceed 0.03–0.04% [52]. The amount of sinapic acid, the specific polyphenol from rapeseed [53], increases in fermented rapeseed meal, comparatively [54]. This fact can be due to the fermentation process that transforms the esterified form into the free form of sinapic acid and this is important if we take into consideration that sinapic acid is a useful matrix for a wide variety of peptides and proteins [55]. Similar results regarding individual phenolic compounds were obtained by some authors [10,11,12]. 

### 4.4. Effects of Fermentation on Carbohydrates and Organic Acids Content

Predictably, fermentation in the presence of yeasts led to a dramatic decrease in soluble carbohydrates. Consumption of soluble carbohydrates during the fermentation process of rapeseed meal is frequently reported in the literature [56]. In our study, the decreases in carbohydrates are much higher than those obtained when applied to an enzyme-based fermentation [57]. The reduction in the non-structural carbohydrates (starch) after fermentation of the rapeseed meal with *Aspergillus niger* observed from 3.71 to 1.22%, is also lower than the reduction observed in our study [41]. 

All the organic acids decreased to various extents, some dramatically (citric, tartaric and malic acid); the only exception was recorded in the case of succinic acid, which increased several times (*p* < 0.001) for both yeasts. Succinic acid is the end product of anaerobic metabolism, derived from the fermentation of carbohydrates and has been used widely in the agricultural, food and pharmaceutical industries. The potential of rapeseed meal for succinic acid production was assessed by simultaneous saccharification and fermentation processes using *Actinobacillus succinogenes* ATCC 55618 and reported a yield of 11.5g/100g dry matter [58]. 

In our study, the increase in succinic acid content for fermented rapeseed samples was lower, as no saccharification was used. However, the fermentation for 24 h still led to a 3.9-fold or 6.9-fold increase (in the case of *Saccharomyces cerevisiae* or *Saccharomyces boulardii*, respectively). Further fermentation for 48 h led to further, yet limited, increases in succinic acid: 12.21% in the case of fermentation with *Saccharomyces cerevisiae* yeast and 77.69% in the case of *Saccharomyces boulardii* yeast. This led to the conclusion that the fermentation with *Saccharomyces boulardii* led to slower accumulation of succinic; however, the extent of accumulation after 72 h was similar for the two yeasts (214.90 versus 229.35 mg/100 g). 

### 4.5. Effects of Fermentation on Fatty Acids Content

Chromatographic analysis revealed that fermentation of rapeseed let to a tendency of increasing the UFA and MUFA/100 g FAME content and decreasing in SFA and PUFA/100 g FAME content.

However, the nutritional importance of the changes induced by the fermentation on the fatty acids profile is limited by the fact that the fat content of the rapeseed meal is low; therefore, having little influence when included in animals’ diets. 

### 4.6. Fermentation Effects on Minerals Content 

There were no significant differences, in the case of macro-elements compositions in the studied rapeseed meal. Both micro-elements Cooper and Zinc are known for their beneficial effects on the immune response of the organism. Except for the Cu and Zn concentrations, our results contrast with those of [37,59] who reported increases in both macro- and micro elements concentration.

## 5. Conclusions

The fermentation of rapeseed meal with either *Saccharomyces cerevisiae* or *Saccharomyces boulardii* yeasts influenced both nutritional and anti-nutritional factors contained by the rapeseed. Our results showed that two of the anti-nutritional factors, intact glucosinolates and 3-butyl isothiocyanate, having a negative effect on animal growth performance, were significantly decreased by 51.60–66.04% and 55.21–63.39%, respectively, by fermentation with either *Saccharomyces cerevisiae* or *Saccharomyces boulardii* for 24 h. Further prolongation of the fermentation for another 48 h brought no supplementary decrease in these anti-nutritive factors. 

The fermentation led to a 10–13% increase in the protein content, with slight variations upon the utilised yeast and fermentation time, which brings the fermented rapeseed meal closer to the protein value of soybean meal, a classical reference for feedstuff. The fermentation doubled the Copper concentration and significantly increased the Zinc concentration by 15% as well as several polyphenolic compounds (sinapic acid, gallic acids) known for their beneficial effects on animal health. 

Overall, the results showed that fermentation with *Saccharomyces cerevisiae* or *Saccharomyces boulardii* reduced the content of antinutritive factors and increased the protein content of the rapeseed meal, without major adverse effects on its overall nutritive value.

These results suggest that fermented rapeseed meal could be a promising feed source enriched in important healthy compounds for piglets after weaning. Further nutritional studies are needed to investigate the different rates of incorporation into the piglet diet and to provide more data about the effect of using fermented rapeseed meal in piglets upon weaning. 

## Figures and Tables

**Table 1:** The total intact glucosinolates and 3-butenyl isothiocyanate content in rapeseed meal samples.

Anti-Nutritional Factors (mg/g)	RSM	FRSM-*SC*		FRSM-*SB*		SEM Time	*p* Time	SEM Yeast	*p* Yeast
	0 h, no Yeast	24 h	72 h	24 h	72 h				
Sinigrin	3.74 ^a^ ± 0.06	1.81 ^b^ ± 0.16	1.74 ^b^ ± 0.13	1.27 ^c^ ± 0.16	1.49 ^bc^ ± 0.04	0.043	0.447	0.053	<0.001
3-butenyl isothiocyanate	16.12 ^a^ ± 0.11	7.22 ^b^ ± 0.98	6.83 ^b^ ± 0.16	5.90 ^b^ ± 0.71	5.98 ^b^ ± 0.16	0.205	0.727	0.252	<0.001

^a, b, c^ Mean values which do not have a common superscript letter are significantly different (*p* < 0.05); SEM time = standard error of the means corresponding to the levels of the “time” factor; *p* time = the significance of the effect of the “time” factor; SEM yeast = standard error of the means corresponding to the levels of the “yeast” factor; *p* yeast = the significance of the effect of the “yeast” factor.

**Table 2:** Proximate chemical composition of the rapeseed meal samples (%).

Chemical Composition (%)	RSM	FRSM-*SC*		FRSM-*SB*		SEM Time	*p* Time	SEM Yeast	*p* Yeast
	0 h, no Yeast	24 h	72 h	24 h	72 h				
Dry mater	90.56 ^a^ ± 0.35	87.56 ^b^ ± 0.86	88.41 ^b^ ± 0.34	90.70 ^a^ ± 1.52	88.37 ^b^ ± 1.78	0.101	<0.001	0.124	<0.001
Crude protein	34.95 ^b^ ± 0.16	39.54 ^a^ ± 0.70	38.85 ^a^ ± 0.26	39.03 ^a^ ± 0.98	38.46 ^a^ ± 0.18	0.164	0.125	0.201	<0.001
Crude fat	1.30 ^a^ ± 0.07	0.89 ^c^ ± 0.17	1.13 ^b^ ± 0.17	1.36 ^a^ ± 0.10	1.18 ^b^ ± 0.16	0.008	0.147	0.010	<0.001
Crude cellulose	10.26 ^c^ ± 0.02	12.54 ^a^ ± 0.14	12.32 ^ab^ ± 0.12	11.95 ^b^ ± 0.07	12.35 ^ab^ ± 0.29	0.058	0.519	0.071	<0.001
Ash	8.040 ^b^ ± 0.14	8.64 ^a^ ± 0.14	8.23 ^ab^ ± 0.45	8.03 ^b^ ± 0.28	8.20 ^ab^ ± 0.05	0.055	0.332	0.068	0.014

^a, b, c^ Mean values which do not have a common superscript letter are significantly different (*p* < 0.05); SEM time = standard error of the means corresponding to the levels of the “time” factor; *p* time = the significance of the effect of the “time” factor; SEM yeast = standard error of the means corresponding to the levels of the “yeast” factor; *p* yeast = the significance of the effect of the “yeast” factor.

**Table 3:** The total phenolic compounds (TPC) and antioxidant activity (DPPH, ABTS, Fe^2+^ chelating activity) in rapeseed meal samples.

	RSM	FRSM-*SC*		FRSM-*SB*		SEM Time	*p* Time	SEM Yeast	*p* Yeast
	0 h, no Yeast	24 h	72 h	24 h	72 h				
TPC	85.38 ^a^ ± 3.84	66.22 ^b^ ± 4.09	66.95 ^b^ ± 2.66	65.30 ^b^ ± 0.82	65.27 ^b^ ± 2.88	0.91	0.866	1.12	<0.001
DPPH	2254 ^a^ ± 0.77	1779 ^b^ ±11.06	1835 ^b^ ±181.19	1779 ^b^ ±49.95	1870 ^b^ ± 141.76	21.95	0.124	26.88	<0.001
ABTS	7319 ^a^ ± 12.20	6169 ^c^ ± 43.54	5807 ^d^ ± 89.01	6180 ^b^ ± 143.26	5258 ^e^ ± 273.02	31.21	<0.001	38.23	<0.001
Fe^2+^ chelating activity (%)	5.59 ^c^ ± 1.98	34.45 ^a^ ± 1.82	24.11 ^b^ ± 1.64	40.93 ^c^ ± 2.15	23.06 ^b^ ± 1.36	0.399	<0.001	0.489	<0.001

TPC–expressed by mg GAE (gallic acid equivalent)/g dry sample; DPPH and ABTS—expressed by µmoli Trolox/100g dry sample; ^a, b, c, d, e^ Mean values which do not share a common superscript letter are significantly different (*p* < 0.05); SEM time = standard error of the means corresponding to the levels of the “time” factor; *p* time = the significance of the effect of the “time” factor; SEM yeast = standard error of the means corresponding to the levels of the “yeast” factor; *p* yeast = the significance of the effect of the “yeast” factor.

**Table 4:** Individual phenolic compounds (flavonoids and phenolic acids) in rapeseed meal samples.

Individual Polyphenol (mg/100 g)	RSM	FRSM-*SC*		FRSM-*SB*		SEM Time	*p* Time	SEM Yeast	*p* Yeast
	0 h, no Yeast	24 h	72 h	24 h	72 h				
Gallic acid	1.32 ^c^ ± 0.27	4.98 ^a^ ± 0.47	4.23 ^ab^ ± 0.61	4.12 ^ab^ ± 0.64	2.29 ^bc^ ±1.03	0.239	0.044	0.293	0.001
Catechin	6.02 ^a^ ± 0.13	4.15 ^ab^ ± 0.39	2.90 ^bc^ ±1.10	1.32 ^c^ ± 0.05	1.13 ^c^ ± 0.23	0.242	0.135	0.198	<0.001
Vanillic acid	29.80 ^a^ ± 0.76	12.55 ^c^ ±2.99	22.61 ^ab^ ±3.55	26.79 ^ab^ ± 0.61	19.59 ^bc^ ±2.76	0.905	0.485	1.108	0.001
Caffeic acid	12.69 ^a^ ± 0.68	5.62 ^c^ ± 0.81	8.51 ^bc^ ± 0.95	10.72 ^ab^ ±1.69	10.69 ^ab^ ± 0.79	0.373	0.122	0.457	<0.001
Epicatechin	51.28 ^a^ ± 0.72	4.76 ^c^ ± 0.62	9.86 ^b^ ±2.03	6.55 ^bc^ ± 1.58	3.79 ^c^ ± 0.08	0.441	0.257	0.540	<0.001
p-Coumaric acid	1.39 ^b^ ± 0.07	3.53 ^a^ ± 0.75	4.27 ^a^ ± 0.24	4.18 ^a^ ± 0.15	4.07 ^a^ ± 0.35	0.144	0.344	0.177	<0.001
Ferulic acid	12.2 ^a^ ± 0.04	0.45 ^b^ ± 0.07	0.24 ^c^ ± 0.02	0.23 ^c^ ± 0.04	0.17 ^c^ ± 0.01	0.014	0.004	0.017	<0.001
Sinapic acid	3.44 ^d^ ± 0.36	22.54 ^b^ ± 0.99	26.13 ^a^ ± 0.40	18.28 ^c^ ± 0.86	20.66 ^b^ ± 0.10	0.228	0.001	0.280	<0.001
Rutin	4.39 ^a^ ± 0.06	0.14 ^c^ ± 0.04	0.31 ^c^ ± 0.01	0.83 ^b^ ± 0.13	0.74 ^b^ ± 0.08	0.025	0.504	0.031	<0.001
Quercetin	1.90 ^ab^ ±1.24	1.07 ^b^ ±1.18	2.64 ^ab^ ± 0.12	3.22 ^a^ ± 0.33	3.35 ^a^ ± 0.31	0.211	0.107	0.258	0.013
Luteolin	1.76 ^a^ ± 0.09	0.82 ^c^ ± 0.14	1.27 ^b^ ± 0.03	0.93 ^c^ ± 0.10	0.79 ^c^ ± 0.09	0.032	0.067	0.039	<0.001
Sum	126.20 ^a^ ± 2.07	60.61 ^c^ ±3.60	82.97 ^b^ ±2.42	77.17 ^b^ ± 0.97	67.27 ^bc^ ± 3.27	1.669	0.396	2.045	<0.001

^a, b, c, d^ Mean values which do not share a common superscript letter are significantly different (*p* < 0.05); SEM time = standard error of the means corresponding to the levels of the “time” factor; *p* time = the significance of the effect of the “time” factor; SEM yeast = standard error of the means corresponding to the levels of the “yeast” factor; *p* yeast = the significance of the effect of the “yeast” factor.

**Table 5:** Carbohydrate content in studied rapeseed meal samples.

Carbohydrate (mg/100 g)	RSM	FRSM-*SC*		FRSM-*SB*		SEM Time	*p* Time	SEM Yeast	*p* Yeast
	0 h, no Yeast	24 h	72 h	24 h	72 h				
Fructose	478.00 ^a^ ± 3.75	182.00 ^b^ ± 0.28	57.05 ^c^ ±23.69	21.50 ^d^ ±6.08	12.10 ^d^ ± 4.96	4.159	<0.001	5.094	<0.001
Glucose	640.00 ^a^ ± 41.46	173.70 ^b^ ± 5.66	135.25 ^b^ ± 45.75	114.71 ^b^ ± 131.10	66.10 ^b^ ± 67.46	25.750	0.456	31.530	<0.001
Sucrose	4512.20 ^a^ ± 12.07	219.10 ^b^ ± 45.68	100.15 ^c^ ± 20.44	9.16 ^d^ ± 4.45	15.27 ^d^ ± 10.23	8.545	0.021	10.466	<0.001
Maltose	60.20 ^a^ ± 15.53	38.23 ^a^ ± 25.55	59.40 ^a^ ± 28.00	0.00 ^a^ ± 0.00	0.00 ^a^ ± 0.00	6.317	0.460	7.737	<0.003
Total	5690.40 ^a^ ± 22.81	613.03 ^b^ ± 25.51	351.85 ^bc^ ± 29.63	145.36 ^c^ ± 141.63	93.46 ^c^ ± 82.65	28.100	0.039	34.410	<0.001

^a, b, c, d^ Mean values which do not have a common superscript letter are significantly different (*p* < 0.05); SEM time = standard error of the means corresponding to the levels of the “time” factor; *p* time = the significance of the effect of the “time” factor; SEM yeast = standard error of the means corresponding to the levels of the “yeast” factor; *p* yeast = the significance of the effect of the “yeast” factor.

**Table 6:** Organic acid (OA) content in studied rapeseed meal samples.

Organic Acid (OA, mg/100 g)	RSM	FRSM-*SC*		FRSM-*SB*		SEM Time	*p* Time	SEM Yeast	*p* Yeast
	0 h, no Yeast	24 h	72 h	24 h	72 h				
Oxalic	567.60 ^a^ ± 1.28	393.70 ^b^ ± 5.52	220.40 ^c^ ± 3.96	164.80 ^d^ ± 1.56	152.50 ^d^ ± 32.95	5.613	<0.001	6.875	<0.001
Citric	254.00 ^a^ ± 5.46	168.20 ^b^ ± 37.90	98.05 ^bc^ ± 18.31	39.90 ^cd^ ± 1.85	3.21 ^d^ ± 21.07	7.852	0.354	9.616	<0.001
Tartaric	452.80 ^a^ ± 3.02	150.15 ^b^ ± 25.24	33.05 ^c^ ± 7.00	7.63 ^c^ ± 0.95	3.56 ^c^ ± 1.94	4.381	0.001	5.366	<0.001
Malic	53.00 ^ab^ ± 3.42	28.50 ^bc^ ± 13.29	18.75 ^c^ ± 2.76	29.75 ^a^ ± 13.79	5.69 ^c^ ± 0.98	3.229	0.007	3.955	0.006
Succinic	32.80 ^b^ ± 15.72	191.50 ^a^ ± 22.20	214.90 ^a^ ± 43.27	129.07 ^ab^ ± 29.18	229.35 ^a^ ± 45.18	12.09	0.052	14.80	<0.001
Total OA	1360.20 ^a^ ± 17.97	932.00 ^b^ ± 53.67	585.20 ^c^ ± 16.67	310.40 ^d^ ± 28.84	495.10 ^c^ ± 45.22	12.95	0.026	15.86	<0.001

^a, b, c, d^ Mean values which do not have a common superscript letter are significantly different (*p* < 0.05); SEM time = standard error of the means corresponding to the levels of the “time” factor; *p* time = the significance of the effect of the “time” factor; SEM yeast = standard error of the means corresponding to the levels of the “yeast” factor; *p* yeast = the significance of the effect of the “yeast” factor.

**Table 7:** Fatty acids content in rapeseed meals.

Fatty Acids (g/100 g FAME)	RSM	FRSM-*SC*		FRSM-*SB*		SEM Time	*p* Time	SEM Yeast	*p* Yeast
	0 h, no Yeast	24 h	72 h	24 h	72 h				
SFA	13.72 ^a^ ± 0.14	13.49 ^a^ ± 0.75	11.62 ^a^ ± 1.89	13.05 ^a^ ± 0.37	12.58 ^a^ ± 0.30	0.366	0.406	0.449	0.237
MUFA	42.15 ^b^ ± 0.07	47.81 ^a^ ± 0.01	46.90 ^a^ ± 1.30	46.34 ^a^ ± 0.07	46.30 ^a^ ± 0.13	0.218	0.344	0.267	<0.001
PUFA	43.68 ^a^ ± 0.05	38.71 ^c^ ± 0.73	40.02 ^bc^ ± 1.13	41.09 ^b^ ± 0.08	40.92 ^bc^ ± 0.16	0.243	0.309	0.297	<0.001
UFA	85.83 ^b^ ± 0.21	86.52 ^ab^ ± 0.74	86.92 ^ab^ ± 0.17	87.43 ^a^ ± 0.01	87.22 ^ab^ ± 0.29	0.163	0.787	0.199	0.005
SFA/UFA	0.15 ^a^ ± 0.01	0.15 ^a^ ± 0.01	0.13 ^a^ ± 0.02	0.14 ^a^ ± 0.00	0.14 ^a^ ± 0.00	0.004	0.404	0.005	0.174
PUFA/MUFA	1.03 ^a^ ± 0.01	0.80 ^b^ ± 0.01	0.85 ^b^ ± 0.05	0.88 ^b^ ± 0.00	0.88 ^b^ ± 0.00	0.008	0.297	0.010	0.000

SFA—Saturated fatty acids, MUFA—monounsaturated fatty acids, PUFA—polyunsaturated fatty acid, UFA—unsaturated fatty acids, FAME—fatty acid methyl esters; ^a, b, c^ Mean values which do not have a common superscript letter are significantly different (*p* < 0.05); SEM time = standard error of the means corresponding to the levels of the “time” factor; *p* time = the significance of the effect of the “time” factor; SEM yeast = standard error of the means corresponding to the levels of the “yeast” factor; *p* yeast = the significance of the effect of the “yeast” factor.

**Table 8:** Macro- and micro- minerals composition of rapeseed meal.

Minerals (ppm)	RSM	FRSM-*SC*		FRSM-*SB*		SEM Time	*p* Time	SEM Yeast	*p* Yeast
	0 h, no Yeast	24 h	72 h	24 h	72 h				
Ca	0.55 ^a^ ± 0.00	0.58 ^a^ ± 0.01	0.55 ^a^ ± 0.01	0.56 ^a^ ± 0.00	0.55 ^a^ ± 0.01	0.003	0.058	0.004	0.125
P	1.38 ^a^ ± 0.02	1.44 ^a^ ± 0.04	1.44 ^a^ ± 0.08	1.39 ^a^ ± 0.01	1.39 ^a^ ± 0.11	0.024	0.963	0.029	0.349
Mg	0.73 ^a^ ± 0.01	0.82 ^a^ ± 0.04	0.88 ^a^ ± 0.11	0.90 ^a^ ± 0.08	0.91 ^a^ ± 0.10	0.029	0.034	0.035	0.621
Na	0.05 ^a^ ± 0.00	0.05 ^a^ ± 0.00	0.05 ^a^ ± 0.00	0.03 ^a^ ± 0.03	0.05 ^a^ ± 0.00	0.004	0.367	0.005	0.414
K	1.38 ^ab^ ± 0.04	1.41 ^a^ ± 0.00	1.32 ^ab^ ± 0.12	1.26 ^ab^ ± 0.07	1.17 ^b^ ± 0.02	0.023	0.139	0.028	0.013
Cu	10.31 ^b^ ± 0.74	23.25 ^a^ ± 0.30	23.74 ^a^ ± 1.11	24.62 ^a^ ± 0.83	24.25 ^a^ ± 0.42	0.167	0.882	0.205	<0.001
Fe	373.20 ^a^ ± 4.52	376.70 ^a^ ± 29.32	395.60 ^a^ ± 2.67	358.00 ^a^ ± 12.82	395.90 ^a^ ± 40.79	9.952	0.228	12.189	0.752
Mn	93.25 ^a^ ± 3.51	97.51 ^a^ ± 1.52	94.51 ^a^ ± 2.72	94.09 ^a^ ± 13.02	86.04 ^a^ ± 1.63	2.330	0.307	2.854	0.396
Zn	117.70 ^b^ ± 0.21	132.60 ^a^ ± 0.31	134.20 ^a^ ± 1.87	130.30 ^a^ ± 4.17	138.90 ^a^ ± 8.07	1.049	0.062	1.285	<0.001

^a, b^ Mean values which do not have a common superscript letter are significantly different (*p* < 0.05); SEM time = standard error of the means corresponding to the levels of the “time” factor; *p* time = the significance of the effect of the “time” factor; SEM yeast = standard error of the means corresponding to the levels of the “yeast” factor; *p* yeast = the significance of the effect of the “yeast” factor.

## Data Availability

Data is contained within the article.
